# Therapeutic arthropods and other, largely terrestrial, folk-medicinally important invertebrates: a comparative survey and review

**DOI:** 10.1186/s13002-017-0136-0

**Published:** 2017-02-07

**Authors:** V. Benno Meyer-Rochow

**Affiliations:** 1 Department of Genetics and Physiology, Oulu University, Oulu, SF-90140 Finland; 2 Research Institute of Luminous Organisms, Hachijo, Nakanogo, Hachijojima, Tokyo, 100-1623 Japan

**Keywords:** Folk medicine, Traditional healing, Entomo-pharmacology, Health, Alternative therapies

## Abstract

Traditional healing methods involving hundreds of insect and other invertebrate species are reviewed. Some of the uses are based on the tenet of “*similia similibus*” (let likes be cured by likes), but not all non-conventional health promoting practices should be dismissed as superstition or wishful thinking, for they have stood the test of time. Two questions are addressed: how can totally different organ systems in a human possibly benefit from extracts, potions, powders, secretions, ashes, etc. of a single species and how can different target organs, e.g. bronchi, lungs, the urinary bladder, kidneys, etc. apparently respond to a range of taxonomically not even closely related species? Even though therapeutically used invertebrates are generally small, they nevertheless possess organs for specific functions, e.g. digestion, gas exchange, reproduction. They have a nervous system, endocrine glands, a heart and muscle tissue and they contain a multitude of different molecules like metabolites, enzymes, hormones, neurotransmitters, secretions, etc. that have come under increased scientific scrutiny for pharmacological properties. Bearing that in mind it seems likely that a single species prepared and used in different ways could have a multitude of uses. But how, for example, can there be remedies for breathing and other problems, involving earthworms, molluscs, termites, beetles, cockroaches, bugs, and dragonflies? Since invertebrates themselves can suffer from infections and cancers, common defence reactions are likely to have evolved in all invertebrates, which is why it would be far more surprising to find that each species had evolved its own unique disease fighting system. To obtain a more comprehensive picture, however, we still need information on folk medicinal uses of insects and other invertebrates from a wider range of regions and ethnic groups, but this task is hampered by western-based medicines becoming increasingly dominant and traditional healers being unable and sometimes even unwilling to transmit their knowledge to the younger generation. However, collecting and uncontrolled uses of therapeutic invertebrates can put undue pressure on certain highly sought after species and this is something that has to be borne in mind as well.

## Background

### Insects as food and medicine

Investigations into the uses of insects and other invertebrates as sources of medicines or integral components of therapies and as sources of nutrition for humans have had rather different histories. Although some earlier detailed accounts of insects as human food were available [1–4], serious interest in researching insects and their potential as an item in the human diet to safeguard future food security did not occur until 1975, when in the journal “Search” of the Australian and New Zealand Association for the Advancement of Science an article with the provocative title “Can insects help to ease the problem of world food shortage” appeared [5]. Ever since then the numbers of publications dealing with food insects and investigations into the consumption of insects by humans have continually been increasing (see review by Evans et al. [6]). Owing to chemical analyses and nutritional assessments, a wealth of data is now available to unambiguously conclude that insects represent a valuable food category rich in minerals and vitamins, protein and easily digestible fatty acids, not to mention roughage like fibres and chitin deemed to be beneficial for gut functionality, e.g. [7–15].

However, insects and other invertebrates, since times immemorial, have not only been used as an item of food, but have also played important roles in the treatment of diseases and other dysfunctions [16, 17]. As of late, pharmaceutical research has become increasingly interested in marine invertebrates as a source of antibiotics and health-promoting substances, e.g. [18–22]. Yet, medically important terrestrial arthropods have not benefitted from this upswing in activity or the current interest in food insects and have received far less attention than the latter. On the other hand reports, dating back to antiquity and containing information on a wide range of medicinally used insects and other invertebrates, actually appear to vastly outnumber those that were dealing solely with food insects. In more recent times, however, the focus on therapeutically useful terrestrial invertebrates, at the expense of numerous other species, has been on honey bees and their honey, e.g. [23–28], maggots involved in wound cleaning, e.g. [29–31], the medicinal leech *Hirudo medicinalis*, e.g. [32–34] and to a minor extent on the once extremely popular blister beetles, also known as “Spanish flies” [35–38].

One reason for this development could have been the realisation that the descriptions of medicinal uses of insects in the older reports, were often attributed to the Doctrine of Signatures (i.e., in the sense of “let likes be cured by likes”) and were therefore dismissed as superstition or outright nonsense. While this is undoubtedly the case for some of the older descriptions of insect or spider-mediated remedies, e.g. [34, 39–43] and the often weird instructions how to carry out the procedures that would ‘heal body and soul’, some of the recommended remedies could well do with some scientific validation.

Some of the first really comprehensive reviews dealing with insects and their roles in folk medicinal practices were those of Keferstein [44] and Jühling [45], both cited in [39, 40]. Since then several more extensive publications, reviewing therapeutically important invertebrate animals have appeared like, to name but a few [42, 46–56] on arthropods and those on snails by Bonnemain [57] and Thomas [58]. Numerous very useful additional articles exist (e.g., [33, 34, 43, 58–65]), but they usually focus even more than the earlier mentioned reviews on specific groups of insects and other invertebrates or restrict themselves to particular geographic regions and their inhabitants [52, 53, 66, 67]. Borne out by “Google searches” which on April 28^th^, 2016 yielded for entries of “entomotherapy” and “medicinal insects” as search words 11,100 and 7,110 respective hits, but for “entomophagy” and “insects as human food” 140,000 and 10,300, respectively, one can conclude that the body of literature on insects as human food nowadays vastly exceeds that dealing with therapeutically used arthropods and other invertebrates.

This is somewhat surprising, given that Meyer-Rochow [68] has noticed that the uses of insects and sometimes their products as part of folk medicinal practices appear to be far more resilient to change than insects used exclusively as food, which is why nowadays arthropod species and other invertebrates used medicinally often outnumber species (or groups of species taxonomically closely related) used as food in countries where insect consumption has altogether disappeared or is limited to a very small number of species. A case in point is South Korea, for which Pemberton [69] lists 12 medicinally used species, without counting centipedes, scorpions and annelids, and North Korea for which Meyer-Rochow [70] lists 13 medicinally used species, but only 3 species used as human food. Corresponding figures by Okamoto and Muramatsu [71] for a pre-World War II Korea are 77 medically important versus 14 edible species.

Other examples would be Japan, where according to Umemura [46] at least 120 species of insects (some major ones illustrated in Umemura’s book) had roles in treating certain ailments, but according to Nonaka [72] only maximally 7 kinds of insects were regularly appreciated as food or China, where according to Ding et al. [54] 58 species were used in treating ailments, but a far smaller number of species groups (i.e., closely related species as members of a single group) were part of the local diet. Even in Europe honey bee products, bee stings, and ants with their formic acid secretions, let alone medicinal leeches, etc. are still being used by traditional healers and homoeopathists, but insects and other arthropods, apart from the very isolated and locally restricted delicacies of the maggot-containing Italian “*casu marzu*” cheese and the German “*Milbenkäse*” (mite cheese), are now no longer consumed by European citizens. Even more obvious was this asymmetry in Europe a hundred years ago, when Netolitzky [39, 40] was able to provide details of at least 30 species representing 12 families of medicinally used Coleoptera alone.

### Medicinal insects and other invertebrates

Even if Google searches for therapeutic and medicinal insects received fewer hits than those initiated for insects consumed by humans (see above), the number of publications and reports one would need to consider if one were to cover the field in its entirety is still staggeringly huge and in fact represents a task impossible to achieve. To nevertheless come up with something useful, selectivity is therefore a necessity. There are several possible approaches to cover the topic. One would be to deal with the various diseases and disorders and a list of their treatments involving insects and other invertebrates. One immediate problem would be to decide whether to include only physically manifest diseases or to also include psychiatric disorders; another would be whether to follow the classification of diseases according to the International Classification of Diseases ICD-10, or to start the list with externally recognizable disorders and then progress to the human body’s interior organs, i.e., starting with the skin and moving inward or perhaps starting with the head and moving down to the toes. An important further difficulty would be that folk medicinal practices in treating similar conditions are often not identical in different regions (cf., [49, 53, 72–75]), a situation reminiscent of the way food acceptances vary and expressed aptly in the saying “one man’s meat is another man’s poison” or conveyed convincingly in the statement “what may be perfectly acceptable to one community, may be outright objectionable to another” [76]. Therefore, to focus on diseases and to report which insects and other invertebrates are being employed in connection with treatments for them, although interesting, would result in a rather confusing, contradictory compilation.

Other possibilities would be to examine the active compounds that therapeutically employed insects and other invertebrates contain and then discuss the animals on the basis of the relatedness of their active ingredients or to deal with the uses of the insects and other invertebrates region by region. However, there are also problems with these approaches, because firstly for many medicinal species we have no information on their active compounds and for vast regions we lack all knowledge of folk medicinal uses of insects and other invertebrates. Therefore, a list based on active compounds or regional uses would possess huge and unacceptable gaps. Secondly, as with variations in the treatment routines by neighbouring communities mentioned in the preceding paragraph, even within a narrowly defined region, we would encounter inconsistencies in the uses of the supposedly health-promoting arthropods and other medicinal invertebrates.

Some investigators prepared lists in which the species were arranged in alphabetical order by scientific or vernacular names [77, 78], but the most meaningful strategy, albeit also with some inevitable shortcomings, would seem to involve the taxonomic approach, in which the phylogenetic position of the invertebrates under discussion would form the foundation on which the review on the medicinal uses of these organisms can rest. The biggest problem with that approach, however, is that such an order would not reflect the degree of therapeutic importance of each taxon, i.e., its ranking, and moreover, since there are many hundreds if not thousands of arthropods that would qualify to be considered for the list (over 500 insects and mite species according to Oudhia [79] are used as traditional medicine in the Indian state of Chhattisgarh alone), it would be an unwieldy and terribly long list.

Finally, as long as no consistent scientific nomenclature is used to name the insects, spiders, snails and other invertebrates used in folk medicines, there would inevitably be identical species with often two, three or more supposedly ‘scientific’ names, some of the latter out of date and some simply misspelled. Wrong identifications of species represent further possible defects. It is for these reasons that in this review only occasionally species names will be provided. Since the relative importance of a taxon (e.g., genus, family, order, class) can to some extent be gleaned from the numbers of therapeutically important species contained in them, their numbers will therefore be one focus of this survey. Another will deal with the most commonly mentioned disorders for which arthropods and other invertebrates can apparently provide relief. The only two lists that will provide some species’ names (as far as they are known) will be Table 1, which deals with non-insect arthropods like chelicerates, crustaceans and myriapods as well as some other invertebrate taxa and Table 2, which is a based on a translation from the list of Japanese medicinally and otherwise noteworthy insects in a book by Umemura [46], which is difficult to obtain as a second hand copy and also not available by way of the internet.

**Table 1:** Therapeutic invertebrates of non-insect affiliations

Taxon	English name	Malady or target treated	Reference
Platyhelminthes & Nematodes			
*Schistosoma mansoni*	Bilharzia fluke	Diabetes 1	[222]
*Ancylostoma* sp. *Necator americanus*	hookworms	Asthma Crohn’s disease	[223–226]
*Trichuris suis*	Pig worm	Ulcerative colitis; inflammation of colon and bowel	[34, 151]
Annelida			
*Eisenia foetida, Lumbricus rubelus*	Earthworms	Secretions influence murine malignant and lymphocyte cell proliferations	[158]
*Pheretima* spp.	Earthworms	Eaten raw to serve as antidote in snake & spider bites Taken orally or mixed with honey to drink in cases of malaria Crushed and applied to red eyes	[73, 77, 119]
*Metaphire houletti*	Earthworm	Fried and oily substances applied externally to a burn	[153]
*Perionyx* sp.	Earthworm	Crushed fresh and resultant juice to drink to fight piles	[153]
*Lumbricus* sp.	Earthworm	Consumed for haemorrhoids, arthritis, earache, to clean obstructions	[78]
*Lumbricidae* etc.	Earthworms generally	Extracts with antibacterial, prophylactic and neuroimmune sytem supporting functions	[152]
	Earthworms generally	Earthworm extracts with antipyretic, antispasmodic, diuretic, detoxic, antiasthmatic, antihypertensive and antiallergenic effects	[155]
	Earthworms generally	Kidney stones, alopecia, jaundice, arthralgia, infections, anticoagulant & antibacterial effects	[34]
	Black earthworm	Schistosomiasis, lumps	[96]
*Hirudo medicinalis*	(Medicinal) leech	Fried in sesame oil and oil applied over penis for stimulation	[113]
*Hirudo medicinalis*	(Medicinal) leech	Abnormal swellings, wound healing, surgery, piles, osteoarthritis, haematoma, anticoagulents, post-phlebitis syndrome, abscesses	[34, 227]
Mollusca: Bivalvia & Cephalopoda			
*Angulus (=Tellina)* sp.	Sea shell	Ground shell as a mild purgative; women’s diseases	[78]
*Mactra* sp.	Sea shell	Acne	[172]
*Loligo* sp. *Octopus* sp.	Squid Octopus	Asthma treatment with tea of toasted cuttlebone or octopi arms	[228]
*Sepia officinalis*	Cuttlefish	Skin & tooth troubles	[78]
Mollusca: Gastropoda			
Gastropoda generally	Snails generally	Skin, analgesic & ischaemia cardiporotective, syncope, mental illness, vertigo, infections, pain	[34, 57, 58]
*Arion hortensis*	Garden slug, swallowed whole	Treatment for gastritis or stomach ulcer	[163]
Unidentified slugs	N American slugs	Ulcers, bronchitis, asthma	[58, 226]
Unidentified slugs	Snail and slug slime	Facial skin lesions, acne, combat wrinkles, reduce pigmentation	[170]
Unidentified slugs	Slug mucus	Dermatitis, inflammation, calluses, wound healing, warts removal	[163]
Unidentified black slug	Black slug mucus	Wart removal	[229, 230]
Unidentified snails	Garden snails	Snail slime application in cases of skin problems and internal for tuberculosis, gastrointestinal conditions, nephritis; hernias, inflammations, colds & coughs, bronchitis, asthma, pharyngitis; snail extracts work antibacterial against *Staphylococcus* spp., *E. coli*, Propionibacterium and *Helicobacter pylori*, scar and wrinkle removal	[164]
*Pila* sp.	Apple snail	Flesh boiled and drunk for bone healing or locally applied; flesh eaten for injuries; flesh eaten raw for gastritis; flesh cooked & consumed form tongue blister; shell roasted and crushed applied to burns	[73]
*Pila globosa*	Apple snail	Flesh cooked and eaten as relief for asthma, TB, stomach disorders, eye problems	[119]
*Helix* sp.	Common snail	Body consumed to treat haemorrhoids & internal ills	[78]
*Helix pomatia*		Whooping cough, chronic bronchitis	[169]
*Semisulcospira libertina*	Black freshwater snail	Ingested as soup for liver and gastroenteric trouble	[231]
*Lymnaea* sp.	Pond snail	Flesh, boiled in water for measles, liver ailments, swellings and sprains	[77]
Chelicerata: Araneae			
Aviculariinae	Bird-eating spiders, “tarantulas”	Homeopathic uses as “mygale lasidora” tincture	[41]
*Grammostola spatulata* *Psalmapoeus cambridgei*	China “tarantula” S. Am.“tarantula”	Analgesic Analgesic	[88, 146]
*Brachypelma vagans*	“Tarantula”	Tarantula-based beverage with spider crushed or roasted and powdered; sometimes rubbed on chest externally	[148]
*Tegenaria gigantea*	Sheet spider	Web’s ashes with honey as aphrodisiac and for muscular dystrophy symptoms	[113]
*Heteropoda venatoria*	Huntsman spider	Dried spider put in orifice to treat ottorhoea	[77]
*Neoscona excelsus* *Argiope pulchella* *Neoscona mukerjei* *Neoscona theis* *Lycosa poonaensis* *Pardosa birmanica* *Pardosa sumatrana* *Artema Atlanta* *Mirpissa decorata*	Orb web spider Orb weaver Orb weaver Wolf spider Pond spider Marsh spider House spider Jumping spider	Spider drug to cure liver enlargement Spider dry powder with various leaf juices to treat bleedings, dry cough, headache Dry powder used in cases of fever in kala-azar, for purgative condition in children, insomnia and blood dysentery Diuretic and purgative condition Spider drug for insomnia Dry powder used in connection with bronchitis, asthma, arterial sclerosis Toothache Powder to improve memory, loss of voice, applied in cases of epistaxis and to remove body fat Spider drug to cure tonsillitis	[149]
Spiders generally	Spider webs	External uses: Removal of warts; to dress wounds and stop bleeding; internal uses: to cure troublesome, obstinate distemper, reduce intermittent fever; to congeal blood after tooth extraction	[16, 41]
	Webs covered in black soot	Used to cover pale spots on people’s black skin in Chad (Africa) or dress wounds (Tanzania: Marusha)	[49]
	Spider webs	Put over boils, postules, ulcers; covering wounds; curing wound following circumcision (Sudan: Dongolawi); as a filter during sucking blood (Kenya: Kuku)	[49]
Theraphosidae	Bird-eating spiders	Toasted powdered hairs mixed with chalk for pemba floor drawings used in magic about spirits and death	[59]
Chelicerata: Scorpionida & Acari			
*Tytius* sp.	Scorpion	Whole animal used to treat its own sting; Crushed scorpion or extract or dried and ground applied to site of sting	[49, 59]
*Tytius discrepans*	Scorpion	Inhibits *Leishmania* spp. in vitro	[34]
*Leiurus quinquestriatus hebraeus*	Scorpion	Consumed for skin problems & haemorrhoids	[78]
*Buthus martensii or Mesobuthus martensis*	Scorpion	Pain killer, convulsions, palsy, stroke, facial paralysis, migraine, lymph damage, tetanus, parotis, oedema, carbuncle; Speech disorders	[69, 70]
*Palamnaeus swammerdami*	Scorpion	Boiled in mustard oil with extract for massage to relieve rheumatic joints pain	[119]
*Ixodes ricinus & I. scapularis*	Ticks	Vascular & thrombotic ills	[34]
*Ornithodoros moubata*	Soft bird tick	Deliberate tick bites to develop immunity against tick-borne fever bacterium or rubbing crushed tick into small skin incision	[49]
*Boophilus microplus*	Cattle tick	Chickenpox	[17]
*Trombidium grandissimum*	Red velvet mite	Malaria, urogenital ills, paralysis, aphrodisiac	[79]
Crustacea:			
*Ocypode quadrata* *Eupagurus* sp. Name not available	Ghost crab Hermit crab Jellyfish crab	Whole animal used for treatment of asthma and haemorrhage in women	[228]
*Paratelphusa* sp.	Freshwater crab	Boiled in water; water to be drunk to fight jaundice; ground up with male banana flower and consumed to healinflammatory glands	[77]
*Ucides cordatus*	Mangrove crab	Crab fat mixed in white wine to treat haemorrhage in women	[228]
*Scylla serrata* and *Penaeus indicus*	Mangrove crab and river prawn	Treatment of old age diabetics and to cure skin disease	[77]
*Cancer pagurus*	Crab	Body crushed into paste, the boiled and drunk to cure jaundice & liver ills; as a tonic	[113, 119]
*Claridopsis dubia*	Mantis shrimp	Tea of powdered animal for asthma treatment	[52]
*Porcellio scaber*	Woodlouse, slater	Oedema	[70]
Echinodermata: Asteroidea			
*Actinopyga agassizi* *Acanthaster planci* *Asterias forbesi*	Starfish	Anti-tumour Anti-viral Anti-inflammatory	[228, 232]
Unidentified	Starfish	Asthma	[96]
Echinodermata: Echinoidea *Mellita* sp., *Echinometra lucunter*	Sand dollar Sea urchins	Tea of toasted whole animal to treat asthma	[18, 228]
Myriapoda: Chilopoda			
*Scolopendra* spp.	Centipedes	Hyperlipidemia, problems of joints, feet, legs, stroke, convulsions, lumps, snake bites, tumours, carbuncles, tetanus, lymphangitis, cough, alopecia, neuralgia, whooping, osteomyelitis, gangrene	[34, 69, 70, 136]
Myriapoda: Diplopoda			
*Tymbodesmus falcatus* *Sphenodesmus sheribongensis* One *Spirostreptid* species	Millipedes	Malaria	[140]
*Archispirostreptus syriacus*	Giant millipede	Removal of unwanted hair from eyelids	[78]
*Tachypodoiulus niger*	Black millipede	Decoction to be taken orally for tuberculosis	[77]
Unidentified	Jongoo (Swaheli) millipede	Dried, used as ash and rubbed into scarifications	Referred to as “Tausenfüßler” in [51], but could be a centipede (“Hundertfüßler”)

**Table 2:** Note that the taxonomic positions and names of some of the insects mentioned in this table are likely to have changed since the original publication in 1943 by Umemura, on which this table and a similar one in German by Schimitschek (1968) are based. However, in order to avoid ambiguity and confusion, it was felt it was best for this translated version to retain the nomenclature used in the Japanese original. Note also that precise descriptions of the various ways that treatments are administered are not given in this translation. Left out are species included by Umemura (1943) to be used only as generally wholesome food items, e.g., pupae of *Psilogramma increta* and larvae of *Dendrolimus spectabilis, Chilo simplex, Schoenobius incertellus* and *Zeuzera pyrina* (Lepidoptera), larvae of *Vespa japonica* and larvae and pupae of 5 species of *Polistes* (Hymenoptera), larvae of *Prionus insularis* (Coleoptera), adults of *Loxoblemus orientalis, L. doenitzi* and *Gryllodes bethellus* (Orthoptera), *Cloeon dipterum* (Ephemeroptera) and adults of various species of cicadas (Hemiptera). That numerous additional species of insects historically found acceptance as traditional food in Japan is apparent from the list given by Mitsuhashi (2008) in his book on edible insects of the world

Classification	Treatment	Additional information
Lepidoptera (Bombycidae)		
*Bombyx mori*	Larvae employed in stopping bleedings, throat troubles, fever and snake bite; Pupae used in connection with throat problems, tuberculosis, kidney problems; Adults to stop bleedings and counter snake bite.	Faeces used in connection with bellyache, intestines, lumbago, cramps, eye infections, gonorrhoea, brain haemorrhage, haemorrhoids
*Antheraea yamamai*	Larvae used in connection with asthma and cramps; Pupae for throat and skin troubles, lumps and cramps;	Cocoon used in connection with bone fractures
*Antheraea pernyi*	Pupae to reduce tumour growths & lumps	Cocoon to treat bone fractures and cramps
*Dictyoploca japonica*	Eggs used in treating skin problems	
*Rhodinia fugax*	Cocoon for tumour & lump reduction	Whooping cough
Lepidoptera (Sphingidae)		
*Theretra oldenlandiae*	Larvae all considered effective in treatments of TB, stomach upsets, umps, tumours and fever as well as snake bites	
*Theretra nessus*		
*Acherontia styx*		
*Pergesa elpenor lewisi*		
*Macroglossa stellatarum*		
*Herse convolvuli*		
*Psilogramma increta*		
Lepidoptera (Brahmaeidae)		
*Brahmaea japonica*	Larvae used in connection with cramps, respiratory and stomach troubles	Larvae can mitigate anaemia
Lepidoptera (Cochlidionidae)		
*Cnidocampa flavescens*	Larvae help against cramps; Pupae and adults considered to have positive effect on vision	
Lepidoptera (Hepialidae)		
*Phassus excrescens*	Larvae used for lung and stomach troubles and snake bite	
Lepidoptera (Psychidae)		
*Cryptothelea minuscula*	Larvae used for toothache and larvae as well as adults for respiratory problems	Also used in connection with anaemia
Lepidoptera (Aegeriidae)		
*Paranthrene regalis*	Larvae used for stomach upsets, cramps and gynaecological issues	Used in cases of diphtheria
Lepidoptera (Cossidae)		
*Holocerus vicarius*	Used in fighting fever and cramps	
Lepidoptera (Papilionidae)		
*Papilio xuthus*	Larvae and adults all used in connection with skin disorders; adults moreover in treatments of lumps and tumours	
*Papilio machaon*		
*Papilio protenor demetrius*		
*Papilio macilentus*		
*Papilio alcinous*		
*Graphium sarpedon nipponus*		
Hymenoptera (Apidae)		
*Apis indica japonica*	Honey used in connection with skin, respiratory, urinary and intestinal disorders, snake bite and rabies; Wax used also in connection with skin and digestive problems and snake bite: Larvae & adults in connection with rheumatism	Honey given in cases of influenza, the common cold and whooping cough; wax for freckles and constipation
Hymenoptera (Xylocopidae)		
*Xylocopa apendiculata circumvolans*	Larvae used in connection with fever and respiratory/lung ailments	Considered also effective in cases of haemorrhoids
Hymenoptera (Vespidae)		
*Vespa auraria*	Larvae used in connection with skin diseases, fever and respiratory problems; Pupae used in connection with whooping cough Nest material for ear, eye and dental problems, skin disorders and cramps	Larval treatment in cases of haemorrhoids
*Vespa mandarina*		
		Nest material considered effective in venereal disease and haemorrhoids
Hymenoptera (Braconidae)		
*Euurobracon penerator*	Larvae used in cases of cramp	
Coleoptera (Cerambycidae)		
*Apriona rugicollis*	Larvae used in connection with lung problems, cramps and palsy	
*Batocera lineolata*	Larvae used to mitigate cramps	Used in cancer therapy and diphtheria
*Chloridolum thaliodes*	Larvae involved in treating smallpox	
Coleoptera (Meloidae)		
*Epicauta gorhami*	Adults used in treatments of hair, skin excretory (kidney) system and rabies	Considered effective in cases of warts
Coleoptera (Cicindelidae)		
*Cicindela chinensis*	Adults used in connection with skin, tumours and gynaecological problems	
Coleoptera (Telephoridae)		
*Luciola lateralis*	Adults used in connection with bleedings, hair and tumours	Considered effective in cases of whooping cough, haemorrhoids and as a tonic
*Luciola cruciata*		
Coleoptera (Hydrophilidae)		
*Hydrous acuminatus*	Larvae used in connection with skin disorders and cramps	Considered effective in cases of whooping cough
*Hydrophilus affinis*		
*Sternolophus rufipes*		
Coleoptera (Dytiscidae)		
*Cybister brevis*	Larvae prescribed in cases of asthma, respiratory and stomach problems	
*Cybister japonicus*		
*Cybister trpuntatus*		
*Rhantus pulverosus*	Adults used in cases of skin disorders	
Coleoptera (Scarabaeidae)		
*Xylotrupes dichotomus*	Larvae involved in treating gynaecological problems	
Coleoptera (Lucanicidae)		
*Lucanus macrifemoratus*	Larvae and adults involved in treatments of gynaecological problems	
*Psalidoremus inclinatus*		
Coleoptera (Gyrinidae)		
*Gyrinus curtus* *Gyrinus japonicus* *Dineutes marginatus*	Larvae and adults both used in connection with lung and stomach problems, fever and cramps	
Diptera (Syrphidae)		
*Eristalis tenax*	Pupae for eye problems. tooth ache, and fever; adults inconnection with cramps	
Diptera (Tabanidae)		
*Tabanus trigonus*	Adults used for eye disorders and tumours	
*Tabanus rufidens*		
*Tabanus chrysurus*		
*Tabanus mandarinus*		
Diptera (Muscidae)		
*Musca domestica*	Larvae used in treating snake bites and fever, gut and stomach problems and eye disorders; Adults in connection with fever, tooth ache and skin disorders	Effective in cases of haemorrhoids
*Muscina stabulans*		
*Fannia canicularis*		
*Calliphora lata*		Used in connection with venereal diseases
Diptera (Ortalidae)		
*Dryomyza formosa*	Larvae used to reduce fever, in cases of snake bite, gut and stomach problems and eye disorders	
Diptera (Culiciidae)		
*Culex pipiens*	Adults used to counteract venereal diseases	
*Aedes japonicus*		
*Aedes albopictus*		
*Anopheles japonicus*		
Aphaniptera (Pulicidae)		
*Pulex irritans*	Adults all used in connection with venereal diseases	
*Ctenocephalus canis*		
*Ctenocephalus felis*		
Anoplura (Pediculidae)		
*Pediculus corporis*	Adults used in cases of venereal diseases	
*Pedicuus humanus*		
Orthoptera (Gryllotalpidae)		
*Gryllotalpa africana*	Adults used to reduce fever, mitigate skin and kidney troubles and fight tumour growths	Considered also useful in connection with venereal disease
Orthoptera (Gryllidae)		
*Gryllus mitratus*	Larvae and adults used for fever and tumour reduction	Adults used for dysentery
Orthoptera (Locustidae)		
*Oxya velox*	Adults used in cases of fever, respiratory, skin and gynaecological problems	Effective in treating cancer, haemorrhoids and anaemia
*Oxya vicina*		
Mantodea		
*Paratenodera aridifolia*	Adults used in treating fever, beriberi, tooth ache, fever, hair and respiratory problems	
*Paratenodera angustiennis*		
*Statilia maculata*		
*Hierodula patellifera*		
*Mantis religiosa*		
Blattaria		
*Blattella germanica*	Adults used in connection with skin and stomach disorders	
*Periplaneta americana*		
*Periplaneta picea*		
*Blatta orientalis*		
Odonata (Libellulidae)		
*Sympetrum darwinianum*	Adults used in connection with throat aches, asthma, tumours and fever	Larvae considered to help against whooping cough
*Sympetrum elatum*	Adults used for asthma	
*Sympetrum croceolum*	Adults all considered effective in cases of asthma	
*Sympetrum frequense*		
*Orthetrum albistylum*		
*Crocothemis servillia*	Adults used for ear, eye, throat and gut problems as well as fever	Used also in connection with diphtheria and cough
Neuroptera (Myrmeleonidae)		
*Hagenomyia micans*	Larvae used in connection with fever, migraine/headaches, beriberi and gonorrhoea; adults considered effective in cases of whooping cough	
Neuroptera (Sialidae)		
*Prothermes grandis*	Larvae used for treating lung, stomach and gut problems	
Plecoptera (Perlidae)		
Perla tibialis	Larvae considered effective in cases of cramps	
Perla tinctipennis		
Hemiptera (Nepidae, Belostomatidae, Aphidiidae)		
*Lacotrephes japonensis*	Eggs used in connection with bleedings, intestinal and uterine problems; adults in connection with cough, dysentery and haemorrhoids	
*Ranatra chinensis*		
*Ranatra unicolor*		
*Kirkaldya deyrollei*		
*Schlechtendalia chinensis*	Larvae used for endocrine disorders	
Hemiptera (Coccidae)		
*Ericerus pe-la*	Wax used to stop bleedings and in connection with lung and stomach problems	Considered effective against warts
Hemiptera (Cicadidae)		
*Terpnosia vacua*	Larvae used in cases of anaemia; Exuviae for ear problems, tooth ache and fever as well as kidney problems and tumours	Exuviae also considered effective in cases of smallpox, coughs and haemorrhoids
*Platypeura kaempferi*		
*Graptopsaltria nigrofuscata*		
*Cryptotympana japonensis*		
*Tanna japonensis*		
*Oncotympana maculaticollis*		
*Meimuna opalifera*		
Thysanura		
*Lepisma villosum*	Adults used in cases of eye disease and kidney dysfunction	Adults also considered effective in cases of paralysis

One final note of caution: to clearly separate medicinal from dietary uses of insects is impossible as insect dishes deemed healthy and supportive of a speedy recovery and therefore administered to a convalescing patient could be classified under both headings. And there are other difficulties: what about the use of insects and spiders in de-sensitization sessions with people undergoing treatment for entomo- and arachnophobias? Equally controversial would be the inclusion of the uses of insects and other inverts in connection with supposedly health-promoting magic, sorcery and witchcraft. Decisions on where to draw the line between them and folk medicinal practices, proper and improper, valid and invalid uses will inevitably be arbitrary. Therefore, this review, except for a few borderline cases and some brief and cursory remarks on them, coupled with references to further reading, will not cover such controversial uses and neither will there be a focus on insects and other invertebrates administered as a nutritious food supplement solely to let a patient gather strength and assist him or her in a speedy recovery. These remarks are not to mean that all non-conventional practices to promote health and well-being should be declared ‘rubbish’ and dismissed, for practices that have stood the test of time and become part of the “common knowledge” of a people need to be investigated [80]. However, that will not be the task of this review.

### Mere medicinal magic or improperly understood traditional curative knowledge?

Lloyd [41] provides various examples of seemingly magical uses of spiders in the treatment of adverse human medical conditions and citing Pliny (first century C.E.) describes how spiders put into some oil and boiled on the fire can remove earache when some of the oil is dropped into the ear. Pliny recommends “to infuse them in vinegar or oil of roses and stamp them and then drop some into the ear with saffron and it will stop the pain”. Lloyd [41] also quotes Trallianus (6^th^ century C.E.), who claims that “the fly catching spider wrapped in a linen cloth and hanged on the left arm, is good to drive away a Quotidian [malarial fever]”, a method reminiscent of that favoured by Koronides, namely spiders “boiled with oil of bay …. if you anoint the arteries of the wrists, the arms and temples before the fit, the fever abates and seldom comes again”. Aetius (about 500 C.E.) is quoted by Lloyd [41] to have recommended a method “for suffocation of the mothers”, a cerate of spiders applied to the navel “and said it did great good”.

Van Huis [49] mentions how hairy spiders, hairy caterpillars and hairy flies were used in different parts of the world to cure baldness and how singing insects like crickets were used to “cure ear and throat problems”. Water beetles as urine inhibitors were mentioned to be used in Kwangtung [49] and dung beetles were turned to in Europe in cases of haemorrhoids [40] and in Thailand to stop diarrhoea [81]. In Zaire salivating insects were used to stop exaggerated salivation in humans, bees trapped and kept humming in a calabasse were meant to cure stuttering, cooked glowworms mixed with ashes from the fire of the home of the patient and then ingested with some water were supposed to chase away nightmares and the consumption of the fattest caterpillars was seen as the method of choice for the treatment of elephantiasis [63].

Although some of these assumed magico-fantastic healing properties of insects and other invertebrates in the examples given above and wormy creatures used in easing pain stemming from a tooth cavity as well as the consumption of stick insects to lose (and that of fat termite queens to gain) weight, could easily be dismissed as folk beliefs steeped in the teachings of Hippocrates’ “similia similibus” (let likes be cured by likes), one example given in Netolitzky [40] shows that even the most ridiculous seeming procedures could possess some merit. Folk belief apparently once dictated that a sufferer from toothache needed to help a large number of different upside down lying beetles to their feet, i.e., turning them the right way up and that this act of kindness would relieve the pain of the inflamed tooth. Assuming that the person following the advice to turn over the beetles would have performed that task with bare fingers, then, it is to be assumed, the fingers would have picked up some secretions stemming from the beetles.

Such secretions, however, for example that of the lady bird beetle *Coccinella bisexguttata*, but also that of other beetle species, do indeed possess mildly anaesthetic properties and fingers to which secretions of this kind adhered brought into contact with the aching tooth, could well have had a real effect, quite apart from the psychological one. In a similar vein one could think of examining claims that the consumption of stick insects leads to weight loss and that dung beetles help in cases of gut problems. As mentioned earlier (and will be elaborated upon in the Discussion), there is no way to clearly separate folk medicinal traditions from mere superstition and ritual practices; the transitions between them are not only smooth, they are actually constantly under revision as to what actually has and what has not a scientific basis and what could be explained rationally.

## Major kinds of largely terrestrial arthropods used therapeutically

### Most commonly involved orders of Insects

As with insects used by humans in their diet, for which Bodenheimer [4] stated that there was not a single taxon that did not have a fancier somewhere in the world, medicinally important insects and other invertebrates, too, appear to possess representatives in almost every taxonomic group. However, having said that, there are clearly some groups that receive the lion share of attention and amongst the insects, if one goes by the sheer number of different species used, Lepidoptera and Coleoptera as well as Hemiptera and Hymenoptera feature most prominently. There are, however, regional differences and while in Japan Lepidoptera are amongst the most popular therapeutically used insects [46], Hemiptera and Orthoptera feature prominently in India [61] and Costa-Neto [52] states for Brazil that “the orders most used are Coleoptera, Hymenoptera, Orthoptera, and Homoptera”.

In discussing which groups of insects are therapeutically most important, the answer depends on whom one focuses on (which ethnic group, which geographic region) and whether one takes the number of species used as a basis, the frequency with which some insect is used or the kinds of illnesses and disorders a particular species (and perhaps its products) is used in connection with. Since ancient times, for instance, the various kinds of honey bee, including their larvae and pupae as well as their honey, royal jelly, pollen, propolis and wax have been used in treating a host of disorders and ailments all over the world. The word “medicine” itself can be traced to the ancient elixir “mead”, the mildly alcoholic drink of water and fermented honey [82]. Bee’s together with their products undoubtedly represent some of the most widespread therapeutically used insects and for this reason have had their composition (e.g., [83, 84] and their venom [85, 86] chemically analyzed and tested for its antibacterial and anti-arthritic properties [86–88].

### Bees, ants and other Hymenoptera

Bees and their brood can be used directly and are known to be highly nutritious and therefore possibly a potent ingredient in convalescence and health promoting diets [83, 89]. Tangsa people of North-East India allow themselves to be stung once a year by a bee “for good health” (Jugli, pers. comm.). Stinging worker bees are also used in another direct way, namely to inject their poison into sufferers of rheumatic pain and arthritis [43, 52, 90, 91]. But they are not the only stinging Hymenoptera, for just like honey bees the stings of mason bees and those of stinging ants [52] are also considered to possess anti-arthritic properties as are the stings of wasps. Dutta et al. [92] have very recently investigated the antioxidant potential of the common wasp *Vespa affinis* and a somewhat painful method to strengthen a flaccid penis, in which a large *Dinoponera* sp. ant stings the glans of the male organ, has been mentioned by Marques & Costa-Neto [93].

Various members of the Hymenoptera are appreciated contributors to medical potions and treatments and according to Costa-Neto et al. [59, 60] make up almost 50% of all medicinally used insects in the armamentarium of traditional Brazilian healers. However, elsewhere they are popular too: in Africa in order to treat a severe headache in children, a crushed wasp is put in oil or water, which is then used as a hair wash or inhalant [49]. Crushed wasps have also been reported from the Tsonga in eastern South Africa to cure boils on a baby’s head [94] and ground wasp abdomens in milk fed to a puppy dog by the Kamba people in Kenya are supposed to make their dogs strong, but people in many African countries believe that wasps can also make them stronger [49]. Umemura [46] mentions wasp larvae in treatments for haemorrhoids and crushed wasps according to Jugli (pers. comm.) are applied to spider bites are used by members of the Tangsa tribe in North-East India.

Numerous species of ants are also used in treatments for a wide variety of diseases like mumps, asthma, insect stings and bites, pain, dizziness, colds, paralysis, impotence, arthritis, rheumatism and chickenpox in Brazil and Colombia [52] and in China ant medicines are prescribed for several liver-related conditions, to increase the number of white blood cells, and to boost sexual stamina in men and women [95]. Van Huis [49] reports that in southern Africa *Oecophylla longinoda* ants can cure asthma or bad cough, while *Oecophylla smaragdina*, prepared as a tonic in North-East India and chemically analysed by Chakravorti et al. [14], are regarded to be effective in cases of hepatitis B and general fatigue [61]. Velvet ants, locally known as “*oncinha*”, are used in treating bronchitis [96]. Iron ants (*Tetraponera rufonigra*) and black ants (*Bothroponera ruficeps*) are used as medication only for cattle, e.g. mithun (*Bos frontalis*), suffering from foot and mouth disease [64]. Larvae and pupae of the common red ant *Myrmica rubra* are thought by Ahom people and other tribals of Assam to increase fertility in humans [97].

Bee products, including those of the stingless meliponine *Melipona, Tetragonisca* and *Trigona* species in South America [59] and Australia [98] will be examined in more detail further below in a separate section headed “Indirect medical uses of invertebrates”. Here it may suffice to mention that bee products have been used in virtually all regions of the world to treat almost the entire spectrum of human ills. For Europe Droege [99] mentions ulcers, eye and throat diseases, respiratory ailments, prostate trouble, dermatological problems, all of which considered treatable by honey. Rubbing the face with a mixture containing resin, detritus and dead *Trigona* bees has been reported for Brazilian tribes as a remedy against acne [59], while Van Huis [49] reports on the honey’s wide appreciation as a health giver in Africa. In Japan, according to Umemura [46], bees and their products are thought to help in cases of freckles, whooping cough and haemorrhoids, the common cold and constipation, while in Korea Pemberton [69] lists weakness, abdominal pain, angina pectoris, dry cough, nasal polyps, stomatitis or gingivitis as treatment targets. For India one can add mitigating effects in cases of sunstroke, spleen and stomach disorders, flatulence, and parasitic worms [61]. Lokeshwari and Shantibala [100], moreover, list anti-tumour, anti-inflammatory and cytotoxic as well as analgesic effects that bees and their products can provide. Comprehensive reviews dealing with the whole range of ailments deemed responsive to treatments with honey, beeswax, royal jelly, propolis and bee venom have been published [28, 44, 91]. In a list that contains 21 diseases categories, Gupta and Stagaciu [28] report, for example, 43 skin, 26 nose, ear and throat, and 24 cardiovascular conditions to respond to apitherapy (Table 3).

**Table 3:** Holistic healing through apitherapy (after Gupta & Stangaciu [28])

Category of medical problems	Number of treatable conditions per category
Allergies	- 4
Cancers, neoplasms & tumours	- 8
Cardiovascular conditions	- 24
Endocrine conditions	- 10
Eye problems	- 12
Fever& other whole body conditions	- 3
Haematological problems	- 4
Immune system disorders/AIDS	- 7
Infections & viral diseases	- 7
Kidney & urogenital problems	- 16
Metabolic & digestive problems/liver	- 15
Muscular skeletal problems	- 4
Neurological diseases/mental problems	- 19
Nose, ear & throat conditions	- 26
Respiratory problems	- 19
Rheumatology	- 23
Sexual disorders	- 6
Skin conditions	- 43
Teeth/dental problems	- 14
Traumas	- 4

### Termites (Isoptera)

There are, of course, insects and other arthropods less commonly associated by Europeans with illnesses and the honey bee’s medicinal role. To people with a European cultural background these less well known invertebrates include species, which are involved in treatments of an almost equally wide spectrum of diseases and disorders that bees and their products are put to use to. I shall start with termites. Termites, their various developmental stages and castes as well as their “product”, i.e., the termite mound and the material it consists of, are widely used therapeutically. According to Cavalcanti Reis de Figueirêdo [101], there are 28 countries in the world with the highest number of 17 in Africa alone, in which members of the insect order Isoptera dominate medicinal roles. Termites as a source of medicines to treat asthma, hoarseness and sinusitis, wounds, malnutrition, and heart condition [51, 52], anaemia and anti-diarrhoea [61], as well as tuberculosis [96] have been mentioned and their chemical contents have been reviewed by Chakravorty et al. [14]. Winged termite castes are used by Mishing tribals of Assam to fight ulcers [97] and soldier caste mandibles ground into a powder are used by the Tonga in Zambia on incisions “made in the skin above the arm muscles” [49].

The use of reproductive castes like termite queens for women as a nourishing snack, possibly providing minerals to the foetus during pregnancy [102, 103], but also given postpartum to increase female fertility and termite kings for men to improve sexual prowess have been described [49], while soldier castes with large and pointed mandibles have been reported to be used to suture wounds [104, 105]. Clay of termite mounds or termite runways on trees is commonly taken by pregnant women in sub-Saharan Africa and termite “mud”, i.e., pulverized mound material, is given to pregnant women to prevent miscarriages [49]. Nonaka [106] describes the use of termite mud by the San people and Kgalagadi as indispensable for brewing and used in place of yeast for making liquor (itself a kind of soothing medicine), while N’donazi of the Central African Republic successfully treat festering sores with a ‘slush’ of crushed soldier termite heads [107].

### Beetles (Coleoptera)

An extremely popular group of therapeutically used insects worldwide are blister or oil beetles of the Meloidae family, famous for containing a defensive secretion known as cantharidin. This substance can affect a multitude of organs and has found widespread use as a stimulant for the urogenital system acting diuretically and often causing painful erections [108]. Oil or blister beetles apart from their fame as aphrodisiacs and the family’s most notorious species known as “the Spanish fly” (*Lytta vesicatoria*), have also been used in treatments to avail kidney pain, rheumatism, gout, ear and tooth aches, snake and dog bite sufferings, to promote wound healing and hair growth [41, 109] as well as, amongst the Tsonga in southern Africa, to even induce abortions [94]. The use of the meloid *Epicauta gorhami* to remove warts has been mentioned [48] and the Chinese blister beetle *Mylabris phalerata* is known to have been used extensively in ancient traditional Chinese therapies [110]. Schimitschek [42] provides an interesting account of the general medical history of meloid beetles and mentions the use of the so-called “*potio antilyssa*”, bought by the Prussian King Friedrich-the-Great, to treat cases of rabies and Van Huis [49] reports uses of meloid beetles in Africa as aphrodisiacs, but also to stimulate urination, to treat venereal diseases and to stop earaches.

In addition to meloid beetles there are numerous other Coleoptera belonging to many different families, which have received attention as therapeutically important species. Cantharid species in connection with skin allergies and a wet paste of the dung beetle *Catharsius* sp. to treat diarrhoea have been mentiond for Nyishi and Galo of Arunachal Pradesh [65]. That the odour of roasted dung beetles can cure mental illness when inhaled is thought by the Tsonga in southern Africa [94] and treatments with dung beetles have also frequently been carried out in connection with haemorrhoids and bowel problems as Netolitzky [40] have reported for Europeans a hundred years ago. The same author mentions the use of crushed *Geotrupes* dung beetle powder blown into the eyes of sufferers from eye diseases. Tenebrionids are implicated in combating scabies, skin disorders and deafness [40] as well as asthma, arthritis [111], TB and sexual impotence, the latter use having spurred the tenebrionid *Palembus dermestoides*’ popular name of “love bug” [52]. Scarab beetle larvae used in connection with liver cirrhosis are known [68], and lucanids, weevils, cerambycids, carabids all contain species linked to treatments of respiratory problems, clots, bruises and ulcers or regarded as aphrodisiacs [52].

Water beetles, including dytiscids and gyrinids are given pseudo-medical roles in many parts of Africa as stimulants for breast enlargements in adolescent girls, into whose nipples these beetles are induced to bite [49, 112], possibly to create a hormonal reaction [112]. Aquatic beetles, however, have also been implicated in treatments of urinary problems [80] and in the case of *Rhantus pulverosus* asthma and stomach complaints [46]. Netolitzky [40] mentioned cases in which gyrinid beetles were given to bulls and stallions as an aphrodisiac. Pemberton [69] reports the use of the dytiscid *Cybister tripunctatus* in treatments of all types of blood stasis and polyuria and enuresis in elderly citizens and children, respectively. Many species of beetles, including, for example, carabids, coccinelids and lampyrids have been involved in roles to reduce tooth and ear aches and to act as diuretic and sexual stimulants (like *Coccinella septempunctata*: [113]). Ladybird beetles have found a use as earplugs amongst the Adi of North-east India (Megu, pers. comm.) and lampyrid fireflies have been used in the Levant to break up kidney stones and to treat haemorrhoids [78], but have been reported to cause blisters when placed behind the ear [40]. The elaterid click beetle *Oxynopterus mucronatus* has been used in Java as an aphrodisiac. It is possible that some of the effects of beetles other than meloid species, nevertheless, stem from secretions and droppings of cantharidin or cantharidin-like substances, which are eagerly sought and taken up by large numbers of insect species termed cantharophiles, species in other words, which themselves are incapable of producing the defensive substance, but are storing it [108].

### Bugs, (Hemiptera, now including the formerly separate order Homoptera)

Groups of insects with long histories of medicinal uses are unsurprisingly those that have close associations with humans and human habitation; the honey bee has been one example. Others include parasites like bedbugs and lice, and flies as well as cockroaches. The two species of bedbug (i.e., *Cimex lectularius* and *C. hemipterus*) are involved in a variety of therapies dating back to antiquity. According to Hoffmann [62] bed bugs were recommended by Dioscurides (First century C.E.) as a remedy to prevent the occurrence of quarternal fevers. They were also thought to help against snake bite, headaches, constipation, ulcers, arthritis and to stop somnolence and hair loss. Taken with some wine or vinegar they were said to induce leeches to stop feeding. Their smell has been linked to the prevention of epilepsy. Perhaps surprising is that tribes in Chhatisgarh (India) are supposed to use bedbug prepared as a paste to be used internally for urinary disorders [61] and that in German pharmacies *Cimex* as a homoeopathic substance, also in connection with urinary disorders, was still available until not too long ago [62]. *Cimex rotundatus*, consumed with a banana, is used by Kaburi and Rongmei tribals of Manipur for fighting piles [97]. Incidentally, to treat jaundice, drinking of water in which crushed head lice were suspended, has been used until today in some parts of Spain [114] and for the Levant Lev [78] reported the use of *Pediculus* sp. to clear urinary tract obstructions.

Although not featuring in the list of insect orders most used in Mexican or generally South American folk medicines, the order of Hemiptera, apart from the bedbugs of the genus *Cimex*, also contain a good number of medicinally important species. Stink bugs (Pentatomidae) are not only widely appreciated as food, e.g. [115, 116], but have also found uses in various folk medicinal treatments, e.g., as anti-congestives [52] and in connection with goitre, paralysis, and urogenital disorders [61], heart problems [52] and colds and coughs in case of the Wangcho people of Arunachal Pradesh, who use the very spicy haemolymph of *Melamphus rubrocinctus* (Jugli pers. comm.) or squashed *Halyomorpha picus, Mictis tenebrosa* or *Antilochus coqueberti* for that purpose [66]. Specimens of the bug *Aspongopus nepalensis*, known locally as the gondibug, have been reported by tribal people of Arunachal Pradesh (India) to sometimes possess psychoactive properties, resulting in hallucinations and anxiety in the consumer [115]. According to Van Huis [49] specimens of the pentatomid bug *Agonoscelis pubescens* are pressed and the extracted oil is used for cooking and to treat the scab disease of camels in Sudan. *Nezara robusta* has a reputation in southern Africa for dealing well with hangovers [116] and male bloodsucker bugs (*Triatoma* spp.), according to Costa-Neto & Melo [117] when freshly crushed and put into a nostril of a blocked nose will unblock it, while made into a tea can be effective in all kinds of heart diseases.

Aquatic bugs like *Belostoma indica, Gerris spinole* and *Nepa cinerea*, given roasted or as a tonic, are considered health-promoting, while the fire or giant red bug *Lohita grandis*, crushed alive and applied to a wound, is used to stop excessive bleeding [61]. The same authors also mention leafhopper adults, crushed live and applied directly to a wound to stop bleeding or treat gonorrhoea and dried and burned with fumes to be inhaled for asthma. Various species of Cicadellidae have uses amongst the Ao-Naga in North-East India in treating skin eruptions, ulcers, urticaria, eye prohlems, deafness with puss from the ear, and for indigestion and breathing problems [118]. The consumption of cicadas by women to have high and clear voices has been reported for several places in Africa [49] and Costa-Neto [52] lists cicada in connection with bladder complaints, migraine headache and ear infections while Chakravorty et al. [65] report that *Tibicen pruinosus* is used by Nyishi women in Arunachal Pradesh when consumed raw to stop their menstrual cycles.

### Cockroaches (Blattaria)

Ubiquitous insect cohabitants with humans are various species of cockroaches and they, too, therefore could be expected to possess numerous therapeutic roles. Costa-Neto [52, 59] briefly reviews their folk medicinal uses in China and mentions, for example, *Eupolyphaga sinensis* and *E. plancyi* as agents in regulating menstruation and *Periplaneta americana* as a species involved in controlling urination, renal colics and asthma. Asthma as a target of cockroach therapy is also mentioned by [59, 61, 100, 119]. Senthilkumar et al. [61], however, also mention that cockroaches are used (crushed live and applied) anaesthetically to stop bleeding, to heal bone fractures, remove swellings, break up retained blood clots and ingested to, fight TB and induce milk production. Drooling amongst Tangsa children in North-East India is thought to stop when they are given an odourless roasted cockroach to eat (Jugli, pers. comm.).

Van Huis [49], citing older reports, provides information on the uses of *Blatta orientatlis* and *Ectobius lapponicus* cockroaches in Europe in connection with stroke treatments and as a decoction against boils, pustules, warts and dropsy and as a diureticum. According to the same author cockroaches find a use for tetanus in Madagascar [49]. To break up stones in the urinary bladder, ash of *Periplaneta americana* specimens in crude liquor is taken internally by some people in South India [113], while Manipuris in India give fried individuals of the same species to bed-wetting children [97]. On the island of Java a child’s persistent cough has been reported to be fought by forcing the child to swallow a large live *Panesthia angustipennis* cockroach [120], cited in [49]. The author of this review was told by Chinese scientists that cockroaches are the basis for a powerful medicine to relieve stomach ache, whereupon he, tongue-in-cheek, replied that he found that very interesting, because he always gets a stomach ache after consuming cockroaches (actually he would not even dream of knowingly consuming a cockroach). Meyer-Rochow [81], citing Rudder [121] mentions that North Australian Aborigines “treat little cuts with a potion of crushed cockroaches, which they put on the wound.”

### Flies and kin (Diptera)

Flies are the fourth group that have been mentioned above as a group of insects with close associations to human abodes and therefore they, too, must be expected to have found roles in the armamentarium to fight ill health. And indeed a variety of Diptera, and especially their maggots, have had a long history of so-called “larval therapies”, in which the insects feed on necrotic tissue, secreting antibacterial chemicals and leaving healthy tissue alone, e.g. [29–31, 43, 52, 122]. Costa-Neto [52] reports uses of the housefly *Musca domestica* to treat eye cysts and baldness, the latter involving “a mass of crushed houseflies (*M. domestica*)” rubbed on the head and Senthilkumar et al. [61] lists *M. nebulo*, an Indian housefly, also crushed live and applied to the scalp, but for the purpose to darken a person’s hair. The same authors credit its larvae with roles in wound healing and in the treatment of osteomyelitis. An easily understandable emetic use of the housefly, swallowed live, has been reported by Dixit et al. [113] for South India. However, it is not only humans that benefitted from maggot treatments as examples demonstrate, in which panniculitis in an aged donkey was treated with maggots [123] and debridement therapy with maggots was successfully used on serious wounds in a horse [124].

### Grasshoppers, crickets and kin (Orthoptera)

The insect order Orthoptera contains numerous examples of therapeutic insects species, most notably the mole cricket with its uses in kidney, ulcerating and fever complaints as well as in connection with venereal diseases [46], fertility [125], cited in [52] and in the case of oily extracts from this insect, external wound healing [126]. Other species of crickets are mentioned in connection with mental development, urinary problems [52] and, administered in roasted form, to promote dieresis [118]. Crushed live and applied, nymphs and adults of various crickets and mole crickets are also used to treat skin diseases, oedemata and wounds [118, 127]. Roasted grasshoppers of the species *Chondracris rosea* are used by members of the North-East Indian Tangsa tribe to fight allergies (Jugli, pers. comm.).

A variety of locusts, grasshoppers and katydids, consumed fried or roasted, are considered to fight anaemia, ulcers and liver disorders amongst the Ao of Nagaland [116, 127]. Hypertension and anuresis in women using *Acrida* spp. and bedwetting and nightmares using *Lamarckiana* spp. grasshoppers can be added to this list of medicinal orthopterans [52]. Bed-wetting treatment involving grasshoppers has also been reported for places in Uganda and Zimbabwe by Van Huis [49], who moreover, reports the use of burned and pulverized *Phymateus saxosus* grasshoppers as a remedy for dental caries, but points out that the medicinal effects likely stem from compounds of the plants that the insects have consumed.

### Moths and butterflies (Lepidoptera)

Lepidoptera, perhaps because of *Bombyx mori* caterpillars’ age-old importance as a food source and supplier of silk fibre in North-east India and East Asia [128], undoubtedly play a far greater role in folk medicines of Asia than elsewhere in the world. Umemura [46] mentions no less than 28 species of therapeutically important moths and butterflies, whose larvae in particular dominate folk medicinal insect remedies in Japan. In contrast Costa-Neto et al. [60] report for Brazil that Lepidoptera make up only 10% of the armamentarium of therapeutically useful insect species and that their main uses are related to asthma treatment and pain reduction and then involve principally sphingids and psychids; the removal of warts can be achieved with the help of larval secretions. In Africa mopane caterpillars, the larvae of the moth *Gonimbrasia belina*, play a huge role as a food item, but for medical uses Lepidoptera appear to play an even lesser role than they do in South America since Van Huis [49] in his comprehensive review on medically important sub-Saharan insects does not mention them at all and Kutalek and Prinz [51] point out that larval and adult Lepidoptera are widely regarded as dangerous, although butterfly cocoons are apparently used in Zaire for ear problems.

Old reports from Brazil, re-examined by Britton [129], suggest that the up to 10 cm long bamboo caterpillars of the pyralid moth *Myelobia (Morpheis) smerintha*, known as “*bicho de taquara*”, were used dried and powdered as a vulnerary (for the healing of wounds) by the coastal Malali people provided the caterpillar’s head was removed. If the head was left intact when the insect was dried, consumers were reported to fall into a kind of ecstatic day long sleep during which they saw “splendid forests, ate delicious fruits, killed without difficulty most choice game”, but apparently the Malalis took care to indulge only rarely in this “debilitating kind of pleasure”. It therefore appears as if the head must have contained a hallucinogenic substance present most likely in the salivary glands.

Nonaka [106] describes how the bodily juices of squashed psychid bagworms are rubbed on the bellies of Kalahari San children, who suffer from gastritis/stomatitis and Nonaka and Toms [94] list mopane caterpillars, ground and mixed in water, as an antidote given to a person to drink (or as an infusion into the anus). Moth pupae, eaten cooked are considered effective in treating shortness of breath and weak kidneys (or in the case of *Antheraea assama* to treat impotence: [118]) and are used to fight diarrhoea (*Antheraea paphia*), relieve flatulence and loosen congestion (e.g., *Bombyx mori*), cure back pain and stomach disorders (*A. roylei* and *Samia cynthia ricini*: [118, 127]. The ash of *Bombyx mori* caterpillars and cocoons is mentioned by Dixit et al. [113] as a rejuvenating tonic and aphrodisiac, while adult *B. mori* males have been used in traditional medicines of South Korea to treat impotence, premature ejaculation, whooping and ordinary coughs, as well as general weakness [69]. Larvae and cocoons of *B. mori* have been mentioned by Lev [78] in conjunction with wounds, throat inflammation and haemorrhoids.

### Insects representing other orders

As indicated already at the beginning, there seems to be no insect order that does not contain at least one therapeutically used representative species. For example members of the order Mantodea, crushed alive, are applied to bruises or consumed roasted are considered to strengthen kidneys and relieve convulsions [61], their egg masses when ground and applied are used to remove head lice [94] and in Japan the species *Mantis religiosa* is involved in treating beriberi, fevers, lung and eye disorders [46]. In Zaire this insect is used to treat epilepsy, while ant lions are considered a remedy for high fever [130] cited in [50]. Ant lions, representing the order Neuroptera are also used for beriberi and additionally for whooping cough and gonorrhoea in Japan [46]. Chinlampianga et al. [77] report that in Mizoram (India) crushed specimens of the ant lion *Myrmeleon formicarius* are applied to warts that have first been pricked to bleed. Crushed stick insects (Phasmatodea) are applied to small wounds by Manipuri tribals [97].

Dermaptera (earwigs), unsurprisingly, are used in cases of earaches [52], while members of the aquatic insect orders Odonata and Plecoptera (i.e., dragon and stoneflies, respectively) are mentioned by Umemura [46] in connection with cramp and asthma treatments and *Crocothemis servillia* adults, moreover, in ear, eye, throat and stomach disorders. Amongst the Hausa and Ngambaye in Africa, however, dragonflies are feared as they are thought to be able to cause epilepsy and disorientation, but among the Nyanja in Zambia the powder of dragonflies applied to the body is thought to let a fighter avoid punches [49]. Tonics prepared from nymphs of the dragonflies *Acisoma panorpoides* and *Aeshna petalura* are used by Ao-Naga people of North-East India as blood purifiers and anaemia treatment [118] and roasted or boiled *Ephemeroptera* spp. nymphs have been reported by Chakravorty et al. [65] to help in cases of stomach upsets. The use of the primitively wingless silverfish (*Lepisma villosum*) of the order Thysanura, has, according to Umemura [46], once been a tradition in some parts of Japan in connection with paralysis and eye problems.

## Indirect medical uses of insects

### Honey and other insect products

What is meant by “indirect medical uses” is obvious when we think of the sugary secretions of certain aphids appreciated as a health tidbit [4], the dried faecal pellets of insects used in pharmacologically active teas [131, 132] and the discarded exuviae of cicadas ground into a powder [70]. The egg cases of praying mantises rather than the producer of the eggs is taken in Korea to boost a man’s stamina and to treat asthma, pain in the feet, skin rashes, paralysis and conjunctivitis [69]. The same author reports the use of cicada exuviae used as a tea to treat hoarseness of the voice, hearing problems and tetanus. In fact discarded insect skins, i.e. exuviae especially of cicadas, but also of other insects (e.g., the exoskeletons of the grasshopper *Tropidacris* sp.: [52]) are widely used in different parts of the world. Teas based on the excreta of phasmid stick insects and *Bombyx mori* caterpillars are also widely used in parts of Asia in cases of asthma, stomach upsets and body pain, e.g. [131] and cramps, eye infections, gonorrhoea, brain haemorrhage and haemorrhoids [46], respectively.

Insect products of great therapeutic value, are, of course, also honey and bee’s wax, both widely recommended as a remedy for stomach or bowel troubles and numerous other conditions (see below). In fact the great many and varied medical uses of all bee products (not just honey and wax, but also propolis, royal jelly, pollen and bee venom) vastly outnumber the direct uses of the bee and, having received considerable attention over the years, this is why some references to the extensive companion literature that exists on honey and other medically useful bee products should suffice [43, 49, 91, 108, 133].

Incidentally, in some parts of Africa, royal jelly is said to be good for adjusting irregular cycles, but pregnant women are not allowed honey in Cameroon as it is feared it could cause abortions [49]. Generally, however the view in Africa according to Van Huis [49], and shared widely with the rest of the world, is that “honey is supposed to solve numerous health problems: malnutrition, gastric complaints (ulcers), colic, constipation (especially in babies), intestinal worms, dysentery, haemorrhoids, liver diseases, influenza, flu and chest problems such as cough, cold, tonsillitis (honey taken with egg yolk in Tanzania, but also in Benin), diabetes, measles, malaria, smallpox, pustules, boils, mouth sores, leishmaniasis wounds, burns from hot water or fire (especially mentioned in Tanzania), vaginal infections, scabies, eczema, post delivery pain, low and high blood pressure, old age, joint problems, impotence, and irritation of the eyes. It also increases general fitness, fights fatigue, increases fertility of women, stimulates lactation after birth, purifies the blood, gives you a better voice, and acts as an aphrodisiac.”

An exception to this almost world-widely appreciated role of honey as a medicinally potent insect product, are members of a Papuan tribe known as the Karam: to them (Bulmer, personal communication) all use of honey is taboo. Why this should be so has never been properly examined. Whether the use of honey, stored in the abdomens of special castes of honey pot ants and highly appreciated by Central Australia Aborigines [134] has any medicinal uses apart from its nourishing quality, is unknown.

### Insect nests and dwellings

It has repeatedly been reported that instead of the insect or insects their dwellings or’nests’ are put to use in traditional medicines. The Poncaré Indians in Brazil dissolve the whole nest of mud wasps in water and then apply the mixture on mumps; they also melt the wax of the nest of stingless bee *Plebeia* sp. in hot urine and give the cooled mixture a diabetes sufferer to drink. To inhale the smoke of the melted wax is supposed to help in cases of stroke [52]. Termite soil alone, because it can be seen as a carrier of medicine, or together with herbs, is in Africa often, for example, in Chad and Uganda, used to put on wounds or an inflammation and nests of mason and dauber wasps have been reported by Van Huis [49] to possess a number of functions when boiled in water whole without the larvae and pupae. The slush made out of the crushed building material of hard termite mounds mixed with water “can be used as a plaster cast around the fractured limb” in Chad, Burkina, and Mali, while the mud of dauber or mason wasp nests can be placed over a swelling or rubbed on the belly to act against pain of the spleen. When the mixture is taken orally, one spoonful a day for a week, and put into nose or ears (three drops at a time) it is meant to cure sinusitis. Using a crushed wasp nest in oil or water as an inhalant or washing the head with it are measures associated with stopping severe headaches in Mali and Mozambique [49].

In Korea whole paper wasp nests can be used [68] in cases of “child fright disease”, arthritis, lymphangitis stroke, recurrent rashes, ‘*tinea capitis’*, mastitis, toothache and intestinal worms. Nests of the potter wasp *Eumenes* sp. have been reported to be used by Mishings, Bodo and other Assamese tribes in cases of headaches and burns and *Oecophylla* nests turned into a paste with water are applied to boils by Meeteis tribals of Manipur [97].

### Insects as substrates for medicinal fungi

A wide range of positive effects is known for caterpillars killed by *Cordyceps* fungi. The shrivelled insect body supports a mature *Cordyceps*, an insect product in the widest sense, which is highly appreciated in many regions of the world as an ingredient to traditional treatments, i.e., medicines, which according to Pemberton [69], are used in particular for stroke and stroke-induced speech problems, but also for cases of headache, convulsion, tremor, tonsillitis or pharyngitis, uricaris, lymphoedema or mastitis, rubella, and tubercular lymphadenitis in South Korea. Meyer-Rochow [70] encountered exceptional interest by North Korean students in the therapeutic uses of *Cordyceps* and the insects that the fungus develops in. Lung problems could be added to the list of ailments supposedly responding to *Cordyceps* treatment in Brazil [52]. The fungus *Beauveria bassiana*, which affects silkworm caterpillars, is another species of insect pathogen used medicinally in South Korea [69] and it is likely that fungi grown by leaf-cutter ants, e.g., *Atta* spp., in underground chambers in South America could also be producers of therapeutically useful compounds.

### Diagnoses based on the presence of insects

Forensic medicine makes use of insects in a variety of cases [52, 135], but the extent to which insects and other invertebrates help a traditional healer in diagnosing a condition is an area severely under-researched. A few examples may serve to illustrate this. The presence of certain flies indicates to Kiriwinia Islanders in Papua New Guinea that a corpse must be nearby; other species of insects are used to judge whether a water is drinkable or not and the presence of head lice on a child’s scalp can provide information on the child’s status of health [81]. Some tribals in Africa recognize diabetes when ants are noticed to feed on a person’s urine [49] and according to Oudhia [79] “the flights of winged adults [termites: added by this author] are considered as a sign of bad period for health. The natives [of Chhattisgarh: added by this author] are advised to the special precaution during this period”. Undoubtedly, a search for more examples should yield interesting results.

## Non-insect arthropods, gastropods and “worms”

### Centipedes and millipedes (Myriapoda)

Another by people with a European cultural background little appreciated invertebrate group with wide therapeutic applications, especially in southern and eastern Asia, are centipedes, in particular species of the genus *Scolopendra* [48, 69, 70], but with the exception of scorpions (see further below), for other non-insect arthropods like millipedes, arachnids and crustaceans (cf. Table 1), there is unfortunately insufficient information to present a “hierarchy of therapeutic usefulness”, but an in vitro modestly cytotoxic alkaloid against the growth of human tumour cells has been known since 1996 from the East Asian medicinal centipede *Scolopendra subspinipes* [136]. As with honey bees the centipede’s therapeutic applications cover a broad spectrum of disorders that range from alopecia and snake bite to tetanus and tumours. To stop a child becoming the sort of man who gets an erection whenever he sees a woman, Central Australian Aborigines have been reported to strike a boy child’s cheeks with a centipede, placing it thereafter on the child’s genital [137]. Dried specimens, pulverized and used as a medicinal powder in the form of a tea or a paste are the most common methods of application, but external uses of lard made by frying large centipedes in vegetable oil, are also known [70]. In many ways the therapeutic uses of scorpions resemble those of centipedes in that either oily extracts are applied externally in cases of rheumatic joint pains [119] or a powder obtained by grinding roasted scorpions is ingested and used internally to lower blood pressure and strengthen liver function [70].

Medically important centipedes, often species of the genus *Scolopendra*, have already been dealt with and antimicrobial peptides and cytotoxic alkaloids are known from them [34, 48, 136]. There are, however, other myriapods, namely millipedes, which have occasionally been mentioned in connection with traditional healing methods. All millipedes are known to secrete defensive benzoquinones or cyanide-derivatives, e.g., mandelonitrite identified by Noguchi et al. [138] from *Chamberlinius hualienensis*. There are other as yet insufficiently known substances in millipedes, which ought to be examined for their potency in fighting disease-causing microbes and there has been a suggestion that the Bobo people of Burkina Faso consume millipedes in order to develop some degree of resistance to malaria [139]. The millipede *Tachypodoiulus niger* as a decoction to be taken orally is known to be used to fight tuberculosis in Mizoram (India) [77].

### Spiders and other arachnids (Chelicerata)

Scorpions and spiders, both capable of delivering venom, have been mentioned a few times and especially spiders seemed to have played an important role in treating medical problems of the ancient Greek [41]. Not long ago a peptide from the venom of a tarantula has been credited with properties that can be used to inhibit atrial fibrillation in humans [140] and the antivenom of the Australian red-back spider *Latrodectus hasselti* has successfully been used by Atakuziev et al. [141] in the treatment of clinical envenomation by the theridiid spider *Steatoda capensis*. In Korea scorpion treatment is used in connection with several medically quite unrelated conditions, e.g., pain, stroke, oedema, tetanus to name but a few [69, 70] and in the Levant scorpions used to be consumed to fight skin problems and haemorrhoids [78]. A high molecular weight peptide isolated from the venom of the Indian scorpion *Heterometrus bengalensis* and, termed “bengaline”, has been shown to exhibit antiproliferative and apoptogenic activity against human leukaemic cell lines [142, 143]. In rats the same substance was shown to possess antiosteoporosis properties [144, 145].

However, while scorpion venom has been analyzed and found to contain numerous valuable bio-active compounds that can explain the Chinese use of scorpion venom in connection with meningitis, epilepsy, stroke and rheumatic diseases, cf. [34], there is almost certainly an enormous untapped potential for the use of the venom of spiders as well as spider enzymes that are involved in the spiders’ extraoral digestion of their prey. Spider webs appear to have far more often been used in folk remedies than the spider itself and apart from its usefulness in covering up blemishes and small wounds, it may well contain bio-active substances possessing analgesic and antibacterial properties. In view of the fact that many stinging insects, e.g., bees, wasps, and some ants are given roles to play in treatments of rheumatism and arthritis, it seems surprising that spider bites do not appear to feature at all in this regard. Tarantula spider toxin, for example, has been credited with a capacity to reduce pain in humans [146, 147] and to be an integral component of zootherapies in Mexico [148]. Perhaps a biochemical comparison between the venoms of stinging insects and biting spiders can provide interesting insights.

Altogether 18 medicinal uses of 9 spider species, administered most frequently as a powder of dried specimens or parts of them, have been reported by Patel et al. [149] from tribal people in Madhya Pradesh’s Ratanmahal region (India) as remedies for haemorrhages, piles, bleeding, coughs and coryza, congestive headache, nostril bleeding, liver enlargement, kala-azar, insomnia, week memory, hoarseness, reduction of excess fat, epistaxis, bronchitis, asthma, tonsillitis, blood dysentery, rheumatism and arterial sclerosis. Discharge from the ear is stopped by Mizoram tribals in India by stuffing a dead and crushed *Heteropoda venatoria* huntsman spider wrapped in soft cloth into the orifice [77].

An indirect product of spiders with medical uses are, of course, spider webs (cf., Table 1). It has been reported that they serve as plugs to stop nose-bleeding, that –in combination with certain plants– they can be incorporated into pills and potions and that bandages and plasters for minor wounds have been in use since ancient times and in many parts of the world, e.g. [40, 48, 149]. Spider webs covered in black soot and placed over pale spots on the dark skin of an African sufferer is another example of an arthropod’s product instead of the arthropod itself being used [49].

### Worms: annelids, nematodes, platyhelminthes etc

The word “worm” covers members of several animal phyla and is therefore zoologically useless, but in terms of folk medicinal practices tolerable. While hardly based on traditional healing methods, modern research credits certain parasitic helminths and nematodes with assisting the immune system of human hosts and reducing incidences of people harbouring these worms to develop atopisms later in life [150, 151]. Earthworms on the other hand have had a long history as components of traditional medicines, in which, for example, ingested in dry form they were used in Iran to relieve jaundice [152], but to fight piles the juice of crushed worms of the genus *Perionyx* was swallowed by the Karbi of Assam [153]. Members of the same tribe applied fried and oily *Metaphire houletti* earthworms to burns to ease the pain and speed up the healing process [153]. Earthworms baked with bread have been used to treat kidney stones and topically applied dead earthworms were used by North American Indians to relieve arthralgia while earthworm powder exhibited positive outcomes in connection with experimentally alcohol-induced liver enzyme elevations in rats [154]. Zhang et al. [155] found earthworms to exhibit antipyretic, antispasmodic, diuretic, detoxic, antiasthmatic, antihypertensive and antiallergic properties. Engelmann et al. [156] and Cooper et al. [157] tested earthworms for antimicrobial and anticancer molecules and confirmed observations by Hrženjak et al. [158] who had shown that substances were present in *Eisenia foetida* and *Lumbricus rubelus* earthworms (like the glycolipoprotein G-90, for example), which could slow down murine in-vivo tumour growth in a dose-dependent manner and obviously possessed anti-neoplastic properties [158]. Cooper et al. [157] also identified anticoagulents and describe earthworm powder mixed with coconut milk as a fibrinolytic agent and a remedy for infections and Li et al. [159] cloned a lumbrokinase gene encoding a blood-clot dissolving protein from *Eisenia foetida*.

The medicinal leech, as a representative of the oligochaet/clitellate order Hirudinida, is of such great importance that even today it is used as part of the modern medical armamentarium [34]. Traditionally this worm has been involved in treatments of abnormal swellings, wounds, piles, osteoarthritis, haematoma, anticoagulents, post-phlebitis syndrome, abscesses and more [33]. A host of therapeutic bioactive substances has been identified, of which hirudin as a specific inhibitor of thrombin is the most important chemical. Topical hirudin as a cream was effective in reducing haematoma symptoms [160].

### Snails and other molluscs

Snails and slug just like insects have a long culinary history with humans and tools to extract the soft parts of land snails through deliberately punched holes in the shells have been identified from human habitations 12,000 years ago in North Africa [161] and archaeological evidence from a site in northern Alabama suggested that 2,500 years BCE the hunter-gatherer population of the New World also consumed gastropods [162]. However as to whether they also used these gastropods in a therapeutic way is difficult to prove, but likely since ancient Greek already possessed traditions to treat inflamed skin with crushed snails [58], and recommended snail mucus against protocoele, pain related to burns and stomach upsets, abscesses and nosebleed [57]. Snails were frequently associated with femininity and fertility and were thought to speed up delivery, fight female scrofula and, following a lengthy preparation with milk, have positive effects on “the spasms of spitting blood accompanying tuberculosis and for the urine ardour of nephritis” [57] in Italy the common garden slug was swallowed whole “as a treatment for gastritis or stomach ulcers” and its mucus rubbed onto the skin helped in treatments of dermatitis, inflammations calluses, acne and warts [163]. Thomas [58] lists numerous other conditions, in which mostly slug or snail’s slime find an application, often as an external dressing, lotion or cream, but sometimes also as slime syrup to be taken internally.

From terrestrial gastropods are known E_2_-prostaglandins of *Helix pomatia* [164] as an effective bronchial relaxant and a lectin with the name “*Helix pomatia* agglutinin” with potential in treating a variety of cancers (e.g., breast, stomach and colon) [165]. Kubota et al. [166] purified and characterized an antibacterial factor from snail mucus and Brieva et al. [167] established the molecular basis for the regenerative properties of the secretion of the garden snail *Cryptomphalus aspersa*. Effects of the secretion on photodamaged skin of human probands showed positive outcomes on coarse periocular and fine facial rhytides [168].

Snail therapies, still widely used in the 19^th^ century, seemed to be in danger of being outphased in the 20^th^ century, but Quevauviller [169] revived interest in snail and slug-based remedies, especially those involving *Helix pomatia*, and provided several formulations, including snail syrup for whooping cough and chronic bronchitis. Modern chemical analyses of snail and slug mucus components [170, 171] have revealed a host of substances, named helicidine, pertussidine, pomaticine, which possess mucolytic and spasmolytic pharmacological activities useful, for instance, in cases of whooping cough and bronchial complaints. That the venom of marine cone and other fish-catching and fish-consuming snails has yielded a huge amount of neurotoxins, some of which with potentially important applications [20], is mentioned here for the sake of completeness, because to deal comprehensively with therapeutically important marine invertebrates would require a separate review.

## Future insect resource availability and use

### Sustainability and ecological impact

A problem, recognized by Ding et al. [55] and echoed by Costa-Neto and Oliveira [59] is that a unrestricted collecting and uncontrolled uses of therapeutic insects and other invertebrates can put undue pressure on certain highly sought after species. An added problem would be (and to some extent probably already is) the trade of invertebrate animal products used in medicines [75]. Mahawar and Jaroli [172] stress that “there is a need to shift the focus from how to obtain the greatest amount of zootherapeutical resources to how to ensure future uses”. In fact, the traditional uses of stag and rhinoceros beetles in Europe, once widespread in the form of magic amulets of adult beetles to treat urinary problems and as pulverized grubs to fight hydropsy or creams against podagra [39], could have contributed to their decline and even disappearance from parts of Europe, although most likely habitat change and use of insecticides could possibly have had a far greater effect. Nevertheless, Kunin and Lawton [173] certainly have a point suggesting that the highest priority for conservation ought to be given to species involved in traditional remedies.

There are, however, other important tasks that must not be neglected and require collaboration between ethnologists and experts of other subjects. For example, we still need information from many more regions and ethnic groups that have been looked at with regard to folk medicinal traditional uses of insects and other invertebrates. This is an urgent necessity as western-based medicines become more and more dominant and traditional healers are often unable and sometimes even unwilling to transmit their knowledge of age-old remedies to the younger generation. Another problem is the dearth of taxonomic experts required to identify therapeutically important species and to shed light on questions related to their life cycle, association with plants as food or shelter, activity peaks, fecundity etc.

### Effective health-promoting compounds identified from insects and other invertebrates

Given their long history as therapeutic agents, insects have to be seen as potential sources for chemical compounds useful as drugs [174]. However, although there are species that synthesize bio-active compounds metabolically in their bodies [108], ultimately the chemicals sought after and deemed responsible for successful treatments stem from the food the insects and other invertebrates in question have ingested (Table 4). Analyses of the food plants of medicinally important insects should therefore accompany chemical analyses of the insects as suggested by Andary et al. [175], cited in [52] and Alves and Albuquerque [176] and compounds identified and likely to be involved in a healing process ought then to undergo clinical testing. Eloff et al. [177], for example, investigated leaf extracts of the plant *Dodonaea viscose* var. *angustifolia* of the Sapindaceae family for their antioxidant and antibacterial properties, because this plant was the sole host for the highly appreciated edible stink bug *Encosternum delegorguei* in southern Africa [116]. Compounds inhibiting growths of various bacteria (e.g., *Staphylococcus aureus, Enterococcus faecalis, Escherichia coli,* and *Pseudomonas aeruginosa*) were found to be also present to some extent in the plant-sucking bug [177].

**Table 4:** Some examples of therapeutically used invertebrates, for whom bio-active substances are known

Species and/or component	Efficacy and properties	Source
Beeswax, honey & royal jelly	Bio-active, oestrogenic and immunosuppressive compounds	[43, 91, 147]
Bee venom	Anti-malignant, anti-inflammatory, anti-arthritic peptides, e.g. melittin	[85, 88, 178, 179]
Lice salivary glands	Medically effective toxins	[182]
Fly maggots	Anti-microbial secretions	[26, 184]
Stinging ants	Antimicrobial, haemolytic, cytolytic, insecticidal compounds, e.g., peptides	[185, 186]
Meloid beetles, e.g., *Mylabris phalerata*	Cytotoxic effect on human mononuclear leukaemic U937 cells and anti-cancer and anti-leismanial activities; apoptosis inducer	[35, 159, 190, 231]
Centipede *Scolopendra subspinipes mutilans*	Peptides with strong antimicrobial and moderate haemolytic activity against human and rabbit red cells	[34, 48, 136, 233, 234]
Chelicerates (e.g., *Heterometrus bengalensis* scorpion)	Apoptogenic, anti-proliferative, anti-osteoporosis substance, e.g., bengalin	[140, 143–145]
Ticks (e.g., *Ixodes* sp.) and leeches	Cell proliferation inhibitors and Anti-coagulants, e.g., hirudin	[32, 160, 198–200]
Earthworms, e.g. *Eisenia foetida* and *Lumbricus rubelus*	Anti-pyretic, anti-spasmodic, diuretic, anti-asthmatic, anti-hypertensive, anti-allergic, anti-neoplastic properties	[156, 158]

Honey, beeswax, and royal jelly have all been thoroughly and repeatedly been analyzed and screened for bio-active compounds (cf. reviews [43, 91]) and compounds effective in wound-healing, burns, various forms of dermatitis and gastroenteritis could be identified from honey and wax. Oestrogenic and immunosuppressive compounds were isolated from royal jelly and composition and action profiles of bee venom have been under close scrutiny [88]. Clinical trials were conducted [178] and Son et al. [85], for example, found up to 50% of melittin in bee venom, a peptide considered to be anti-malignant and effective as an anti-inflammatory and anti-arthritic agent [179]. Other beneficial compounds that were identified included some that had nociceptive, analgesic and anti-allergic properties. Anti-diabetic and anti-dandruff activity was recently demonstrated by [180]. Antimicrobial peptides, however, are not confined to insect venoms, but have also been identified from numerous insect larvae [181] and anti-coagulants are known from the salivary secretions of blood-sucking ticks and leeches [32]. Even human lice [182] had their saliva scrutinized and tested positive for the presence of medically potentially important toxins.

Following the recognition of the usefulness of fly maggots in treating wounds, burns, osteomyelitis and ulcers, including one that involved methicillin-resistant *Staphylococcus aureus* [183], the anti-microbial activity of maggot secretions was studied by Thomas et al. [26] and Steenvoorde & Jukema [184]. Venoms of stinging ants were analysed by Aili et al. [185] and found to exhibit antimicrobial, haemolytic, cytolytic, paralytic, insecticidal and pain-producing pharmacologies. Earlier Veal et al. [186] had already shown that secretions from the metapleural glands of the Australian bulldog ant (*Myrmecina gulosa*) possessed antimicrobial properties.

Health problems in humans and domestic animals caused by blister beetles (e.g., toxicosis) and staphylinids of the genus *Paederus* (e.g., dermatitis) have been known from many countries and the responsible species were justifiably given the epithet “medically important” by Nikbakhtzadeh & Tirgari [187]. Compounds of the staphylinid *Paederus*, known as pederins, were analyzed by Frank and Kanamitsu [188] and found to contain toxic haemolymph amides with two tetrahydropyran rings. Meloids like the Chinese *Mylabris phalerata*, the European Spanish Fly *Lytta vesicatoria* and the North American species of the genus *Epicauta* secrete mono-terpenes, which are generally referred to as cantharidin. Cantharidin was found to act as a phosphatase inhibitor and to alter vascular endothelial cell permeability [189] as well as exert an anti-leishmanial effect [190]. An effect of cantharidins on leukaemic stem cells has been reported by Dorn et al. [191] and that cell cycle arrest and apoptosis can be induced oxidative stress-independently in pancreatic cancer cells through cantharidin could be shown by Li et al. [159]. The derivative norcantharidin has received considerable attention as an inducer of apoptosis ever since Peng et al. [35] had reported this, possibly through caspase activation following cytochrome C release [35]. In human colorectal cancer its anti-angiogenesis activity has been linked to an endothelial pathway [192].

Bioactive compounds isolated from earthworms, gastropod secretions and spider and scorpion venom have already been briefly mentioned in their respective paragraphs earlier in this review. A high molecular weight peptide, for example, isolated from the venom of the Indian scorpion *Heterometrus bengalensis* and, termed “bengaline”, has been shown to exhibit antiproliferative and apoptogenic activity against human leukaemic cell lines [142, 143], while in rats it was shown to possess antiosteoporosis properties [144, 145]. A tarantula was credited with properties that can be used to inhibit atrial fibrillation in humans [140] and to act as pain killer in humans [146, 147].

Active *substances* in leeches to prevent blood coagulation, treat osteoarthritis and other ailments have received considerable attention and a vast amount of literature dedicated to leech therapy exists, e.g. [193–195]. Earthworms, however, tested by Engelmann et al. [156] have also received attention, especially for antimicrobial and anticancer molecules. Hrženjak et al. [158] were able to show that substances were present in *Eisenia foetida* and *Lumbricus rubelus* earthworms (like the glycolipoprotein G-90, for example), which could slow down murine in-vivo tumour growth in a dose-dependent manner and obviously possessed anti-neoplastic properties. Aanticoagulents were identified by Cooper et al. [157], who also described earthworm powder mixed with coconut milk as a fibrinolytic agent and a remedy for infections, while Li et al. [196] cloned a lumbrokinase gene encoding a blood-clot dissolving protein from *Eisenia foetida.*

Chemical analyses of snail and slug mucus components [170, 171] have revealed a host of substances, named helicidine, pertussidine, pomaticine, which possess mucolytic and spasmolytic pharmacological activities useful, for instance, in cases of whooping cough and bronchial complaints. An effective bronchial relaxant in the form of E_2_-prostaglandins was discovered by Pons et al. [164] in *Helix pomatia* an and a lectin with the name of “*Helix pomatia* agglutinin” appeared to possess potential in treating a variety of cancers (e.g., breast, stomach and colon) [165]. Kubota et al. [166] purified and characterized an antibacterial factor from snail mucus. Cyanogenic compounds of gomphodesmid and benzoquinone containing spirostrepsid millipedes are thought to provide some protection against malaria to the Bobo people in Burkino Fasa, who consume these myriapods [140]. The cyanide-derivative mandelonitrite was analyzed by Noguchi et al. [138] from the polydesmid millipede *Chamberlinius hualienensis* and probably provided that species with protection against predators and possibly microbial attacks during mass migration and cluster formation [197].

Observations that the saliva of ticks, e.g., *Ixodes scapilaris* and *Boophilus microplus*, functions as an inhibitor of angiogenesis [198] and that the tick-derived substance *‘ixolaris’* “may block tumor growth of human cell models with ectopic FVIIa expression through inhibition of direct TF-FVIIa-PAR2 signaling” [199] underscore the conclusion that a huge number of bioactive proteins would be present in sanguivorous animals [200]. However, no such activity was found to be associated with the saliva of *Anopheles gambiae, An. stephensi, Lutzomyia longipalpis, Phlebotomus papatasi, Aedes aegypti, Culex quinquefasciatus,* and *Cimex lectularius* [198].

A common component of the cuticle of arthropods, but also found in some molluscs, helminths and minor animal phyla, but not vertebrates, is the polysaccharide chitin. Its anti-tumour and anti-viral properties have received considerable attention in the past, but its immunological effects are less well known [201]. Chitin can strengthen the immune system as shown with mice by inducing an accumulation in the tissue of IL-4 expressing innate immune cells (including eosinophils and basophils) associated with allergies [202]. The macrophages, stimulated by chitin, produce cytokines and other compounds that are deemed responsible for the observed host resistance to bacterial and viral infections and the already mentioned anti-tumour activity [203]. In the murine model an intranasal spray of chitin microparticles was reported to down-regulate allergic reactions to mite and fungi [203].

## Discussion

### The “Doctrine of Signatures”: let likes be cured by likes

When noisy cicadas are considered to be effective in cases of hearing problems, sanguivorous tabanids are used to treat blood problems and multilegged, fast running centipedes in Korea are the preferred treatment for “leg, foot and joint problems” [69], one cannot help but to invoke the “Doctrine of Signatures”. Folk logic must have dictated that similar appearances and behaviours were indicative of relationships, which were not coincidental, but based on true sameness. A healthy function in one should therefore be able to rub off on another less healthy individual. Although one would probably discard the notion that medicines derived from dung beetles are the perfect remedy in cases of bowel trouble and that stick insects can help an obese patient to lose weight, one should never jump to conclusions prematurely without an investigation illuminating various angles of a historically accepted practice [80, 204].

A widely popular folk medicinal treatment of rheumatic and arthritic pains, for example, involves pain-causing arthropods that sting: scorpions, bees, wasps, ants. It is therefore not possible to easily argue away the thought that the concept of “let likes be cured by likes” is behind this. However, a host of bioactive substances have actually been identified in particular from the venoms of scorpions and honey bees and it is more than likely that all stinging arthropods possess pharmacologically bioactively useful chemicals in their venoms, which can ameliorate rheumatic and joint pain [43, 52, 53]. To what extent they really tackle the causes of rheumatism and how they help mitigating arthritic pains, what the venoms’ chemical structures are and which characteristics of them physiologically affect the recipient, all that needs to be objectively assessed, even if subjectively there is overwhelming acceptance of the effectiveness of such kind of entomotherapy.

However, if a majority of patients accepts a treatment as effective in one place, what might be the reason for the observation that traditional treatments of identical diseases can be very different in two geographically adjacent communities (unpublished observation by the author: Kiriwina, Papua New Guinea) or, on the other hand, can be very similar in completely different areas? Van Huis [49] surmises the latter situation could be due to “migration of people in history, communications ….or local genesis based on observation and discovery”. However, even if migration and communication were involved, at the beginning there must have been observation and discovery and, thus, a likely positive outcome of the treatment. But what if neighbouring communities employ different traditional methods in connection with the same disorder? One possible reason could be that having one’s own remedy, separate from that of the neighbours, aids in the cohesion of a group, helps that group stand out amongst others, assists that group in maintaining its ‘uniqueness’ and creates a feeling of “belonging”. It is therefore not much different from food taboos, which can also vary tremendously, even if they involve the same species, between closely neighbouring groups [76].

### For each malady one species or one species for all ills?

One common problem that doubters have with regard to the validity of most insect, spider, and gastropod treatments is that one organism is supposed to be good for a multitude of illnesses (Table 5). How can that possibly be? The bee and its products that are deemed effective in cases of ulcers, eye and throat diseases, respiratory ailments, prostate trouble, dermatological problems, etc. are just one example, cf. [44, 99]. Meloid oil or blister beetles, e.g., *Lytta vesicatoria*, are another, e.g. [34, 42], but one could equally well choose the stink bug, the garden snail or the cockroach, in fact almost any therapeutically used species appears to have multiple uses.

**Table 5:** Major categories of therapeutic uses of invertebrates (based on a variety of published sources and observations by the author during field work in North-East India)

	Used fresh and crushed or as a paste	Used dried, fried or as a powder	Boiled and use of extract of insect or spider	Tonic or decoction of insect or spider	Other, e.g. smoke & fumes or wash
External: applied to aching/injured body part	19	3	3	--	2
Internal: inhaled, or swallowed with or without liquid	8	25	11	13	6 (as fumes to be inhaled)

How could totally different organ systems in the human body benefit from the extracts, potions, powders, secretions, ashes or whatever of a single species? Although this seems at first glance very problematic, one needs to remember that even though most of the therapeutically used invertebrates are small creatures, they nevertheless just like bigger animals, possess organs in their bodies that are specialized to carry out specific functions, e.g. digestion, gas exchange, and reproduction. The little animals have a nervous system, endocrine glands, a heart and of course muscular tissue.

Obviously in their small bodies a multitude of very different, yet distinct, molecules like metabolites, enzymes, hormones, neurotransmitters, etc. exist and bearing that in mind, it does not seem so unlikely any more that a single species prepared in different ways (and applied externally or given internally to act upon different human disorders: Table 5) could indeed combine in itself a multitude of uses. Invertebrates can get sick (viz. the “Journal of Insect Pathology”) just like vertebrates and publications devoted to treating insects and other invertebrates exist, e.g. [205, 206]. Apparently, in the words of Solter [207]: “Most pathogens, parasites and other aetiological agents of disease in invertebrate animals will be familiar to vertebrate pathologists and, indeed, many if not most are genetically related to disease agents in vertebrate animals.”

Another at first glance rather astonishing observation is that looking at some common human ailments like arthritis and rheumatism or target organs and systems, e.g., the respiratory system with bronchi and lungs, the urinary system with bladder and kidneys, one notices that a wide range of taxonomically not even closely related species are credited with positive effects when used in connection with a particular disorder or organ. How can it be that there are remedies for asthma involving earthworms, molluscs, termites, beetles, cockroaches, bugs, and even dragonflies as well as starfish and how can urinary problems become ameliorated by folk treatments with the same assemblage of unrelated species? In the same vein: how is it possible that the “help” of so many totally different invertebrates is enlisted in combating tumours? According to Oldfield [208] 4% of the extracts from 800 species of terrestrial arthropods, including crustaceans, spiders, insects, centipedes and millipedes) exhibited some anti-cancer activity.

I think the fact that so many taxonomically not at all closely related species can be used to fight one and the same disorder or disease (Table 6) need not be a contradiction. Diseases are often based on the same essentials: pathogenic agents like viruses, bacteria, fungi etc. and in the cases of cancers there are always mutated, rapidly proliferating cell lines involved. All invertebrates can, of course, suffer themselves from infections and even cancers and to fight the disease certain unifying principles are likely to have evolved in all invertebrates [209–211]. It would therefore appear to be far more surprising to find that each group or even each species had evolved its own unique defence system fighting disease. A case in point, although not involving an insect-derived medicine or treatment of a specific condition, is the use of the locked mandibles of insect species belonging to four very different orders of insects: termites, certain ants, carabid beetles, and larval neuropterans known as ant-lions (and maybe even more species of additional insect orders), but they all perform the same task. All these taxonomically not closely related insects use the same biting mechanism and are put to use as surgical micro-clamps around the edges of a narrow wound, a cut perhaps.

**Table 6:** Major disease categories and insect and other invertebrate taxa containing species used in treating diseases of the various categories

	Heart & blood	Skin & wounds & burns	Lungs & bronchi & asthma	Kidney & urogenital	Ulcers & tumors	Haemorrhoids	Muscle & rheumatism	Fever & colds
Odonata & plus aquatic orders	X		X	X	X			X
Orthoptera		X	X	X	X		X	
Isoptera	X	X	X	X	X			
Hemiptera	X	X	X	X			X	X
Coleoptera	X	X	X	X	X	X	X	
Lepidoptera	X	X	X	X	X		X	X
Hymenoptera	X	apitherapy	apitherapy	apitherapy	apitherapy		X	X
Diptera	X	X						X
Other orders	X		X	X				X
Chilopoda	X	X			X		X	
Scorpiondae	X					X	X	
Oligochaeta	X	X	X	X	X	X	X	X
Gastropoda	X	X	X	X		X		X

### A brief look at the vertebrate situation

A survey and review of therapeutically important invertebrate would be incomplete if a quick glance at the situation involving the vertebrate pharmacopeia were not included. Not only have vertebrates and medicines derived from them played important roles in Latin America (e.g., [212–215]) and elsewhere in the world, e.g. North-East India [216] and Africa (e.g., [217, 218]), they have also received considerably more attention than remedies based on invertebrates [219]. Medicinal vertebrates actually feature more prominently than invertebrates as objects of trade at local and traditional markets even in urban settings [213, 220]. Mostly comprising species of reptiles, birds and mammals, these medicinal vertebrate animals number nearly a hundred species, many of which are considered vulnerable and endangered [212–216]. This is why the need has been expressed to comprehensively investigate the bioactivities of the compounds championed by knowledgeable locals as effective in cases of, for example, diseases of the circulatory system [213], so that replacements could be produced [221]. Alternatives to the use of wild animals, it is believed, could then perhaps help protecting threatened faunal elements and lead to a more sustainable use of the resource that wild animals undoubtedly represent.

These developments that are taking place in connection with vertebrates should not be ignored by ethnobiologists involved in the medicinal uses of invertebrates. As modern neither vertebrate nor invertebrate-based medicines are becoming ever more popular and traditional healing methods in some societies are in danger of disappearing altogether, there is on the one hand the need to preserve the knowledge of effective and time-honoured traditional healing practices involving invertebrate species, but this need, as with the vertebrates mentioned above, has to be balanced on the other hand against the danger of wild species becoming over-exploited. Although there appears to be far less trading of medicinal invertebrates than vertebrates going on worldwide (despite some traditional pharmacies selling insects and insect products being quite popular especially in Asian countries), there are also still regions, in which the use of invertebrates to treat illnesses and symptoms not only continues, but actually increases due to an increase in population. As with the vertebrate example given above, developing replacements of the products derived from the invertebrates captured in the wild is one way; another is to find ways to culture the therapeutically most effective and most sought after invertebrates. However, whether customers and traditional healers would then be prepared to lessen their dependence on wild specimens and accept the cultured ones in preference to the former, is another question, which only future can tell.

## Conclusion

The fact that many remedies seem to subscribe to the tenet “let likes be cured by likes” should not necessarily lead us to dismiss or belittle a treatment method that for centuries might have been an accepted way to confront a disease. Unbiased investigations can bring to light truths in what is often referred to as “common knowledge” by traditional folk, but ridiculed as “old wives tales” by modern scientists [80]. The reader is reminded of the narration given in the introduction of this article, in which Netolitzky [40] explains that the “good deed” of turning as many as possible different upside-down beetles back into their normal stance would mitigate toothache, could have a scientific and logical background. How can that be? In the process of touching the beetles many of them are likely to secrete mildly anaesthetic substances that cling to the fingers of the tooth ache sufferer. Unbeknownst to him or her, his or her fingers may from time to time have touched the sore tooth, whereby the anaesthetics from the beetles’ secretions could have exerted their effect. Although admittedly a far-fetched story, it is not altogether impossible that this could have been the origin of the accepted “treatment”, but what this example nevertheless illustrates is that explanations for accepted practices should be sought even if they sound like pure superstition.

Having said all that, I do not wish to be misunderstood as someone who believes that there must always be some truth in the traditional healing methods. That is certainly not correct and some traditional healing methods involving insects and other arthropods could either be outright dangerous (one could think of some of the uses of cantharadine-containing beetles or highly venomous scorpions and spiders) or be based on irrational beliefs and wishful thinking and, thus, be dangerous by preventing a patient from getting proper help. Perhaps the best understood and medically accepted forms of traditional treatments are those involving maggots and leeches. Honey as a bee product is certainly uncontroversial, given proper controls, but in using bee and other insect-sting treatments one must, of course, rule out atopic reactions, which traditional healers may not always do. Nonetheless, I hope this article has shown that traditional healing methods are worth taking a closer look at and that at least some of them in combination with modern methods can indeed improve the outcome of a treatment. The challenge is to identify those traditional healing methods that do have something to offer before nobody knows anything anymore about them and such healing methods have disappeared from the collective memory of a people.

## Abbreviations

AIDS: Acquired immune deficiency syndrome
BCE: Before Current Era
CE: Current Era
FAO: Food and Agriculture Organization
TB: Tuberculosis
